# The relevance of sperm morphology in male infertility

**DOI:** 10.3389/frph.2022.945351

**Published:** 2022-08-03

**Authors:** Elena Moretti, Cinzia Signorini, Daria Noto, Roberta Corsaro, Giulia Collodel

**Affiliations:** Department of Molecular and Developmental Medicine, University of Siena, Siena, Italy

**Keywords:** assisted reproduction technologies (ART), human sperm, sperm morphology, transmission electron microscopy, oxidative stress, genetics, systematic sperm defects

## Abstract

This brief report concerns the role of human sperm morphology assessment in different fields of male infertility: basic research, genetics, assisted reproduction technologies, oxidative stress. One of the best methods in studying sperm morphology is transmission electron microscopy (TEM) that enables defining the concept of sperm pathology and classifying alterations in non-systematic and systematic. Non-systematic sperm defects affect head and tail in variable ratio, whereas the rare systematic defects are characterized by a particular anomaly that marks most sperm of an ejaculate. TEM analysis and fluorescence *in situ* hybridization represent outstanding methods in the study of sperm morphology and cytogenetic in patients with altered karyotype characterizing their semen quality before intracytoplasmic sperm injection. In recent years, the genetic investigations on systematic sperm defects, made extraordinary progress identifying candidate genes whose mutations induce morphological sperm anomalies. The question if sperm morphology has an impact on assisted fertilization outcome is debated. Nowadays, oxidative stress represents one of the most important causes of altered sperm morphology and function and can be analyzed from two points of view: 1) spermatozoa with cytoplasmic residue produce reactive oxygen species, 2) the pathologies with inflammatory/oxidative stress background cause morphological alterations. Finally, sperm morphology is also considered an important endpoint in *in vitro* experiments where toxic substances, drugs, antioxidants are tested. We think that the field of sperm morphology is far from being exhausted and needs other research. This parameter can be still considered a valuable indicator of sperm dysfunction both in basic and clinical research.

## Introduction

Among animal species, the man shows a relatively low fertility and, in fertile individuals, a percentage of altered, immotile and dead spermatozoa is present (1). The first scientist who described in the seventeenth century the spermatozoa and their morphological variability was Anton van Leeuwenhoek and since then, the study of sperm morphology became more and more relevant, particularly in this era when infertility is a real medical and social issue.

Over the years, the sperm morphology evaluation has become much more severe, indicating that a careful analysis of this parameter plays an important role in routine semen analysis (2). The reference value for normal sperm morphology, reported in the different editions of World Health Organization (WHO) guidelines for semen analysis, sharply decreased from ≥ 80.5% reported in the 1st edition (3, 4) to ≥ 14% in the 4th edition (5). They lowered up to ≥ 4% in the 5th and 6th editions (2, 6). These observations support and confirm that the man produces a high proportion of defective sperm compared to other animal species.

With the introduction of assisted reproductive technologies (ARTs), sperm morphology evaluation became a cornerstone for prognostic purposes. However, in most cases the analysis was and is still limited to the assessment of normal sperm percentage at light microscopy (LM) level (7). The sperm morphology evaluation by LM is easy but shows technical limitations in terms of resolving power and is unable to spot alterations of structures as chromatin texture and axoneme. Therefore, the “best-looking” spermatozoon by LM could not be lacking morphological defects (1, 8–10). Many efforts have been made in improving sperm morphology evaluation and many other methods were used to enhance the observation of the inner regions of spermatozoon using, for example, high magnification methods and polarized light (11–13).

The questions on the relevance of sperm morphology in determining male fertility potential (14) and its impact on ARTs outcome are debated. While previous data showed a predictive role of sperm morphology in the reproductive outcome, recently acquired data seems not to confirm this (15). Many studies reported the genetic origin of different sperm alterations. In rare situations, most sperm show a specific defect as in globozoospermia or dysplasia of fibrous sheath (DFS) (3, 16) and, in these cases, it is easy to recognize and quantify it. However, little attention is still paid to the quantification and the description of human sperm abnormalities that represent mixed combinations of head and tail defects found in variable percentages in different ejaculates (7, 9, 17).

Many limitations in sperm morphology assessment are still present and are mainly due to the physiological variability of most morphological sperm characteristics, the subjective nature of the evaluation, the utility of this parameter in the choice of an adequate treatment of the patient. This brief report deals with the role of human sperm morphology in different fields of male infertility: basic research, genetics, assisted reproduction technologies, oxidative stress.

## Sperm morphology at transmission electron microscopy levels

One of the best methods to study sperm morphology is transmission electron microscopy (TEM) that enables the evaluation of the cellular inner organization at high magnification discriminating the normal and altered structures. TEM is useful in the assessment of sperm defects that can influence the fertilizing potential. Ultrastructural studies combined with immunocytochemistry and molecular techniques (1, 9) overcome the description of morphology and allow a detailed characterization of sperm defects from structural, molecular, and functional points of view (18). These last methods enabled the definition of the sperm pathology concept that was important for the classification of ultrastructural alterations in “non-systematic” and “systematic” sperm defects (1, 19).

### Non-systematic sperm defects

Non-systematic sperm defects are common alterations of head, connecting piece and tail structures combined in variable ratio in semen samples. They increase in presence of andrological disorders such as infections and varicocele (20), other endogenous and exogenous factors (21–23) and they may respond to different treatments. In the last decade, TEM analysis was useful in the study of sperm chromatin vacuoles that can be present in multiple copies per cell. An association between large vacuoles and sperm chromatin immaturity has been demonstrated (24). Recently, the focus was oriented on the sperm centriolar region, since, in humans, sperm itself contributes centrioles to the zygote (25, 26). Fishman et al. (27), using electron microscopy with high pressure freezing, demonstrated that in mature human spermatozoa two centrioles are present, revising the centrosome reduction dogma (28). The proximal centriole showed the typical barrel shape and the distal one was composed of splayed microtubules surrounding luminal proteins. Another poorly explored structure is the centriolar adjunct, a sort of “mini flagellum” originating from proximal centriole, visible by TEM in spermatids but partially or completely disappeared in mature sperm. Recent studies reported the presence of centriolar adjunct increased in length in spermatozoa of infertile patients (29) and in a patient who produced aneuploidy embryos with both natural fertilization and ARTs (30).

**Figure 1:**
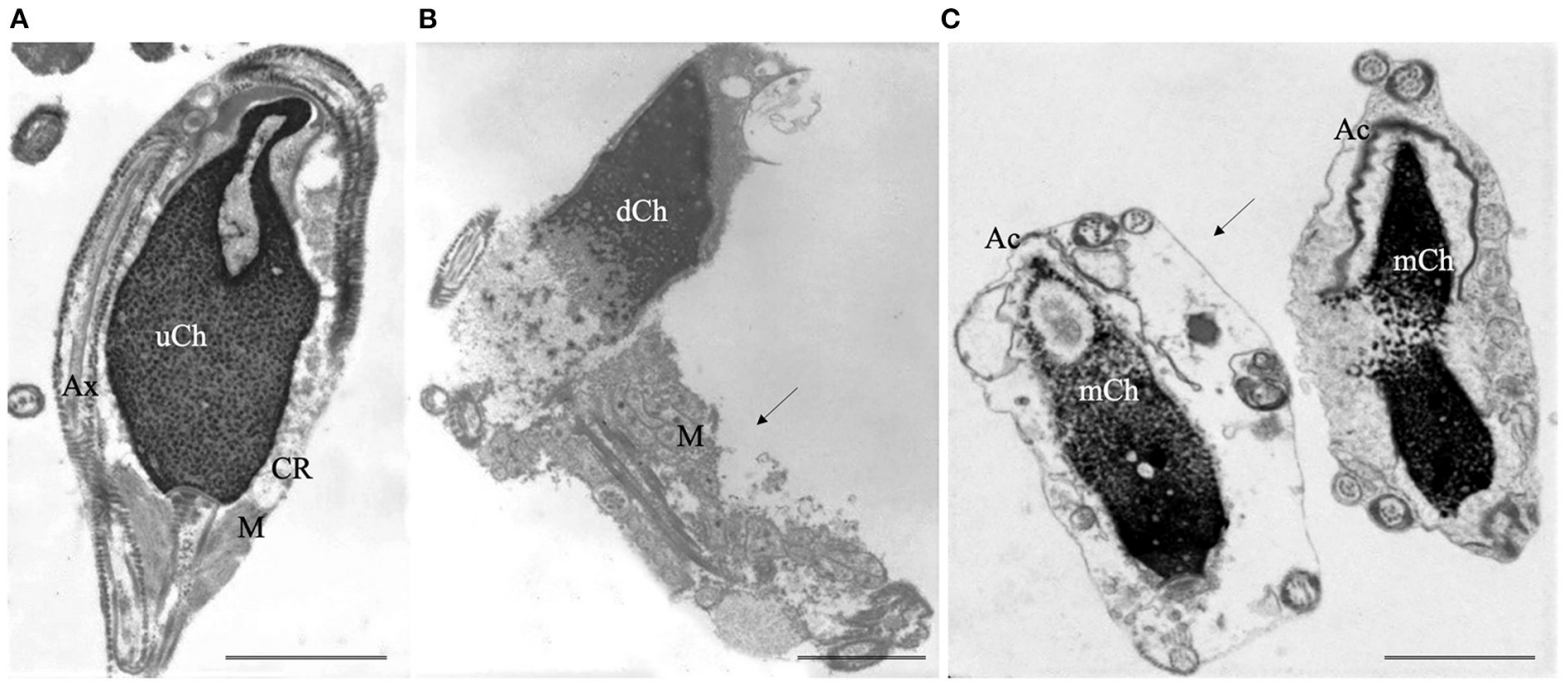
Transmission electron microscopy (TEM) micrographs of longitudinal sections of immature **(A)**, necrotic **(B)** and apoptotic sperm **(C)**. Immature sperm **(A)** is characterized by irregular nucleus with uncondensed chromatin (uCh). Cytoplasmic residue (CR) embeds swollen mitochondria (M) and coiled disassembled axoneme (Ax). Necrotic sperm **(B)** shows an altered nucleus with disrupted chromatin (dCh), swollen mitochondria (M) and broken plasma membrane (arrow). In figure **(C)** two apoptotic sperm with marginated chromatin (mCh), acrosome (Ac) far from the nucleus, integer plasma membranes (arrow) are shown. Bars A, B, C: 2μm.

TEM analysis is mainly a qualitative method and statistics applied to TEM examination of ultrathin sections are questionable, particularly in long cells as spermatozoa, due to the impossibility of determining whether the observed sections belong to the same or different sperm cells. Accordingly, our research group (17, 31) developed a Bayesan formula that quantifies the data obtained by TEM observations. The formula calculates the number of spermatozoa without ultrastructural defects (the fertility index) and the percentages of sperm pathologies as immaturity (Figure 1A), necrosis (Figure 1B) and apoptosis (Figure 1C). The considered ultrastructural characteristics concern acrosome (position, dimension, shape, and content), nuclear shape, chromatin texture, centrioles, mitochondria, axonemal and periaxonemal structures, plasma membrane, and presence/absence of cytoplasmic residue. This quantitative method enabled the comparison of sperm quality in different categorized patients (20, 32, 33) and before and after a treatment or therapy (34, 35). It is still used in research and validated by many publications over the years.

### Systematic sperm defects

Systematic sperm defects are rare alterations characterized by a predominant anomaly present in most sperm of an ejaculate and similar in all patients with the same condition (1, 19). Sperm head, connecting piece and flagellum are regions that can be affected by systematic sperm defects. Since these alterations show family clustering and are significantly more frequent in individuals with a history of consanguinity, a genetic origin was supposed (19). In recent years, the field of genetic investigations related to systematic sperm defects has made extraordinary progress. These studies allowed the identification of candidate genes whose mutations induce morphological sperm anomalies, enhancing the knowledge of sperm pathophysiology important for improving patient management.

The most frequent systematic sperm defects are reported here.

**Figure 2:**
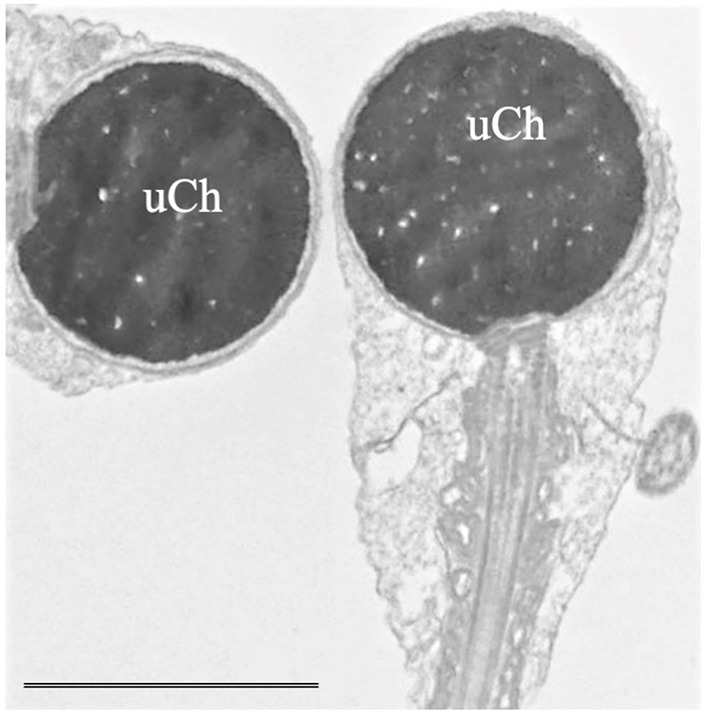
Transmission electron microscopy (TEM) micrograph of sperm with globozoospermia. The round-shaped heads were devoid of acrosome, the nuclei show uncondensed chromatin (uCh) characterized by granular texture. Bar: 3.5 μm.

**Figure 3:**
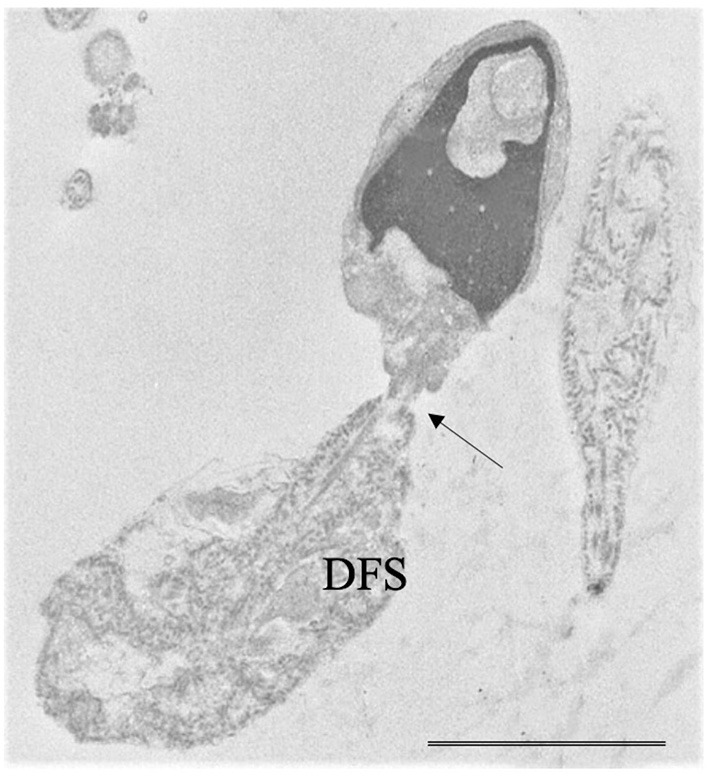
Transmission electron microscopy (TEM) micrograph of a sperm with a combination of flagellar alterations: hyperplastic fibrous sheath (Dysplasia of fibrous sheath, DFS) and almost detached tail (arrow); in addition, altered nucleus and acrosome are visible. Bar: 3.5 μm.

- “Globozoospermia" is characterized by sperm with round head acrosome-less spermatozoa with immature chromatin (Figure 2), depletion of the oocyte activator phospholipase Cζ, located in the inner acrosomal membrane and in perinuclear theca, that causes problems in ICSI outcome (16, 36, 37). Many putative genes, studied also in animal models, seem to be mutated in globozoospermia and many of them encode proteins involved in acrosome biogenesis (36). Although pathogenic variants in DPY19L2 and SPATA16 are known causes of globozoospermia and explain up to 70% of all cases, other candidate genes such as ZPBP, CCDC62 (38), and SPACA1 (39) were recently identified.
- “Macrozoospermia” is characterized by large-headed spermatozoa with multiple flagella (36, 37) and is often due to mutations of Aurora Kinase C (AURK) gene that ensures efficient meiosis (37, 40, 41).
- “Head-tail detachment” / “acephalic sperm” shows an extreme fragility of the connecting piece (42–44). Since centrioles are linking the sperm head with the tail, the mutations of proteins in the centriolar area can cause head-tail disengagement (25) leading to the acephalic sperm phenotype. Recently, Nie et al. (43), based on TEM observations, classified in different subtypes the broken points of sperm connecting piece in acephalic sperm. The subtype one, which etiology needs to be explored, is characterized by sperm head with proximal centriole and tail with distal centriole; in the subtype two, the head contains both centrioles and the identified genes involved are HOOK1, SUN5 (44) and PMFBP1. In the subtype three, the sperm tail contains both centrioles and TSGA10 and BRDT are the involved genes (43).
- “DFS” / “Multiple Morphological Flagellar Anomalies” (MMFA) is characterized by severe asthenzoospermia with almost zero progressive sperm showing short, thick, and irregular tails and disorganized fibrous sheath (36, 42, 45, 46). In the past 5 years, 18 genes whose mutations cause MMAF were identified (46) even if about half of MMAF individuals remain with unknown genetic causes. The analyzed genes encode for outer and inner dynein arms (DNAH1 is one of the most important), for proteins that are involved in connecting axonemal and periaxonemal structures (CFAP43 and CFAP44), in radial spoke complex and centrosome (46).
- “Primary Ciliary Dyskinesia (PCD)” is a heterogeneous disorder characterized by asthenozoospermia or total sperm immotility generally concomitant with bronchitis and rhinosinusitis and airways infections, due to the common presence of the axoneme in cilia and flagella. To date mutations in over 40 genes have been identified, but the exact effect of these mutations on spermatogenesis is poorly understood. Most of these genes encode proteins of outer and inner dynein arms, dynein regulatory complexes and axonemal organization and some of them are in common with MMAF (47). At LM the sperm flagellum shows normal length with no motility. TEM analysis allows the direct observation of the absence of outer/inner, or both dynein arms, of one or both central microtubules; alterations of radial spokes, transposed microtubules, the lack of axoneme, alterations often present in PCD (47).
- Very rare combinations of the above systematic sperm defects such as alterations in head-tail attachment and DFS [Figure 3, (48–50)], the concomitant presence of PCD and DFS (51), globozoospermia and head-tail detachment (52) were also reported in literature.
- The identification of mutations causing systematic sperm defects and the correct genotype–phenotype association by TEM assist in the prognosis of intracytoplasmic sperm injection (ICSI) outcome.

## Sperm morphology and aneuploidies

Fluorescence *in situ* hybridization (FISH) requires probes, complementary to DNA sequences, directly labeled with or detected by a fluorochrome. This technique allowed the study of human sperm chromosomes.

A recent review concerns the impact of sperm parameters on incidence of numerical sperm abnormalities. Revising the 18 years' experience on FISH applied to spermatozoa, the authors concluded that only sperm concentration has negative correlation with aneuploidies (53). However, the topic is debated since many papers reported a relationship between sperm morphology and a slight increase of aneuploidies. Sarrate et al. (54) observed that some of the most predictive variables for altered sperm FISH results are oligozoospermia and altered sperm morphology. These sperm aneuploidies could play a moderate negative impact on embryo quality, implantation, and pregnancy rates.

For this purpose, the studies on the relationship between sperm aneuploidies and recurrent pregnancy loss (RPL) are of particular interest. Focusing only on sperm morphology, many observations agree with a positive relationship between abnormal sperm morphology and increase of sperm aneuploidies that can be one of the possible causes of unexplained RPL (55–58). In most studies, the sperm morphology was evaluated at LM level, which is the widely used method with low magnification power. Collodel et al. (59) analyzed a group of male patients with RPL who showed normal semen parameters, including sperm morphology, at LM levels and found an increase of sperm aneuploidies associated with sperm apoptosis detected by TEM. The relationship between sperm morphology and aneuploidies is interesting also for systematic sperm defects such as in case of macrozoospermia caused by AURKC mutations (40). In this case, large-headed spermatozoa with double or triple nuclei (60) have an abnormal DNA content mainly referred to polyploidy. De Braekeleer et al. (61), revising studies on macrozoospermia, reported that in 30 analyzed males over 90% of spermatozoa were aneuploid, mainly diploid, and characterized by increased DNA fragmentation.

Even though the relationship between globozoospermia and increased sperm aneuploidies is debated (61, 62), the current idea tends to a slight increase of aneuploidies in this defect. The results concerning systematic defects affecting sperm flagellum and aneuploidies are very scant. Several studies have demonstrated an association between DFS and increased frequency of sperm aneuploidies (45, 63–65). Recently, Wambergue et al. (66) reported that 6 infertile patients with MMAF due to homozygous DNAH1 mutations showed an increased frequency of XY and 18 disomy, however no differences respect to control for chromosomes 13, 21, and XX and YY disomies were found.

Due to the reduced number of cases available, it is difficult to draw conclusions, but it is evident that aneuploidy results for the same chromosomes are variable in different reports from different research groups, probably due to technical aspects and a possible inter-individual variability, which is worth exploring.

**Figure 4:**
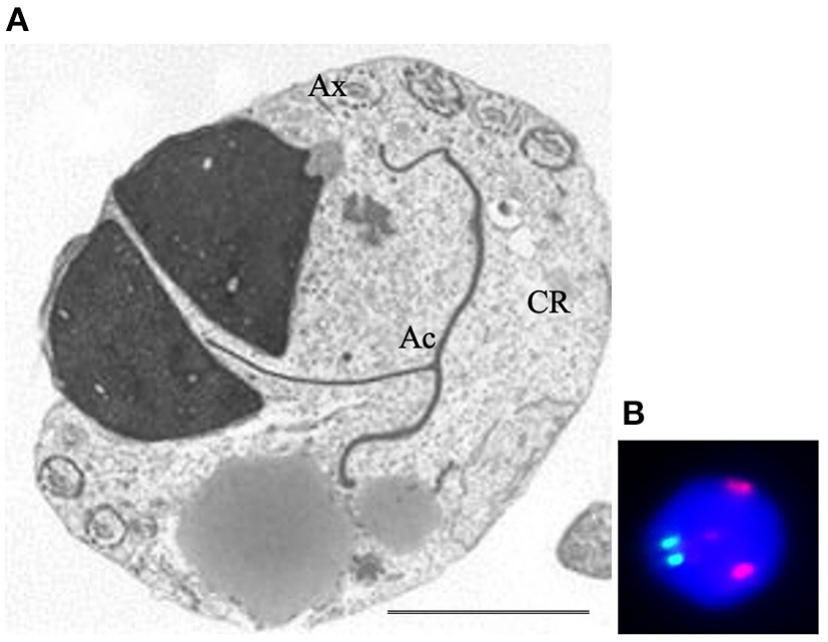
Transmission electron microscopy **(A)** and UV **(B)** micrographs of binucleated, diploid sperm. Figure **(A)** shows two nuclei embedded in a large cytoplasmic residue (CR) where the tail (Ax) is coiled. The acrosome (Ac) is mislocated. Figure **(B)** shows fluorescence *in situ* hybridization with probes for chromosome 18 (green) and 9 (red). The nucleus is diploid since shows two spots of each chromosome. Sperm nucleus is stained with DAPI (blue). Bar A: 3.5 μm.

Another field of research concerns the study of sperm parameters, including morphology and aneuploidies in patients with altered karyotype. The most common karyotype abnormalities in infertile men include numerical sex chromosome alterations and Robertsonian translocations (67). FISH coupled with TEM analysis could represent outstanding method in the study of sperm morphology and cytogenetic in patients with altered karyotype and several papers from our and other groups were published during the first decade of the 2000s. Overall, the sperm pathologies found in spermatozoa, in carriers of reciprocal and Robertsonian translocations (68–72), were immaturity and apoptosis concomitant with increased frequency of diploidy and disomies which are variable among different carriers. A common characteristic was the presence of spermatozoa with two (Figure 4A), three or multiple nuclei that showed a severely altered chromosomal constitution (Figure 4B).

Increased incidence of apoptosis, detected by TEM, and diploidies were observed in spermatozoa from a group of chromosome 9 pericentric inversion carriers (73). These kinds of studies were also performed in sperm of a man with a 46 XY, 47 XY+18 mosaic karyotype; also in this case sperm apoptosis and immaturity were associated with aneuploidies of chromosomes 18, X and Y (74).

The impact of karyotype alterations on male infertility can be faced by many points of views: how the chromosomal anomalies influence sperm parameters, ART outcome, embryo quality and miscarriages (75). In addition, chromosomal alterations, in particular Robertsonian translocations, can interfere with the segregation of other chromosomes, phenomenon known as interchromosomal effect (76, 77).

Nowadays, the procedures of sperm selection for ICSI cannot assure the choice of a spermatozoon without chromosomal imbalance, neither analyzing sperm at high magnification with Nomarski optics (78). Therefore, it is important to improve the studies of the relationship between sperm morphology and chromosome alterations for a correct definition of sperm quality before ART.

Recently, Chen and Zhou (79) published a retrospective study/systematic review on the association between autosomal reciprocal translocation/Robertsonian translocation and semen parameters, topic debated in the literature. They concluded that the carriers of translocations had decreased sperm parameters including sperm morphology as emerged reviewing previous studies.

As perfectly summarized by Ioannou et al. (80), some undoubtable findings are present in the literature: some chromosomes are more prone to non-disjunction, all men produce aneuploid sperm, sperm aneuploidy frequency is increased in infertile men and is correlated to the severity of infertility.

## Impact of sperm morphology in assisted fertilization

The question of the impact of sperm morphology on ART outcome is debated; despite previous data showed a predictive role of sperm morphology in the reproductive outcome, recent findings seem not to confirm this. It is reputed that the main issues on evaluation of sperm morphology are related to the lack of consensus on the classification method: the inter-observer variations, the manual assessment, the subjective nature of morphological evaluation, the use of different staining methods and preparation of smears as well. Thus, the current trend is neglecting the influence of abnormal sperm morphology in the selection of a particular ART (15). Indeed, Del Giudice et al. (81) reported that semen quality, including morphology, is not associated with pregnancy rate.

The weakness of the evaluation of sperm morphology at the LM is related to the poor resolving power, making this method, alone, inadequate to precisely define the morphology of a spermatozoon. Cassuto et al. (82) proposed a scoring scale for sperm morphology, detected at high magnification, useful for ICSI protocol and demonstrated that sperm with severely altered nucleus showed chromatin decondensation, underlining the importance of the way the morphology is evaluated (83).

The introduction of ICSI, the outstanding technique applied in ART (84), bypassed the natural selection barriers between the oocyte and the sperm and raised many ethical and evolutionary concerns. Therefore, many efforts have been made in improving sperm morphology evaluation for the selection of the spermatozoon to be injected during ICSI. One of the most important ideas was represented by the analysis of sperm at high magnification. Therefore, “Motile Sperm Organelle Morphology Examination” (MSOME) was proposed; this method allows the assessment of nucleus, nuclear vacuoles and the selection of a good quality sperm that can enhance the ICSI outcome (11, 85, 86). By the integration of MSOME analysis and ICSI, the Intracytoplasmic Morphologically Selected Sperm Injection (IMSI) was introduced. The method was promising but the results were conflicting (87). Recently, Dieamant et al. (88), revising the literature, found that IMSI appeared to be an effective procedure in reducing the congenital malformations in newborns compared to ICSI.

The use of polarized light applied to the optical microscope/micromanipulator represented another strategy, based on morphological characteristics, to improve the ICSI sperm selection (89), assuming that the spermatozoon shows a natural birefringence due to the configuration of its different components. The natural sperm birefringence represented a good indicator for the ICSI outcome (90–92), but unfortunately, as for IMSI, the equipment is expensive and more studies on DNA integrity should be advisable. Many other techniques for sperm selection in ART, that considered sperm membrane properties, the ability to bind hyaluronic acid or based on microfluidics (selection performed on size and motility of sperm) are available but none of them has shown outstanding results in term of pregnancy rate (87, 93).

It is well known that most laboratories perform the tests manually, with a consequent observer variability during the analysis. Currently, computer-aided sperm analysis (CASA) is a common automated system for routine semen analysis in animals and humans. However, there are many concerns on completely bypassing the human operator, since CASA is still inaccurate in the evaluation of sperm morphology (94). Technological advances such as the application of artificial intelligence (AI)-based devices promise improving the efficiency of the analysis and the reliability of the results (94). For this purpose, an emerging and attractive field on human reproduction and embryology is represented by the use of AI, machine learning and deep learning. These technologies are potentially applicable to many aspects of reproductive medicine, as sperm, oocyte and embryo selection, prediction of ART outcome, semen and sperm morphology evaluation (95–98).

## Sperm morphology and oxidative stress

In recent decades, much evidence has suggested the central role of OS in the etiology of male infertility. A variety of factors can lead to the generation of reactive oxygen species (ROS) in the male germline: unhealthy lifestyle (smoking habit, alcohol abuse, psychological stress) environmental factors (high temperature, metal, and plasticizers exposure), environmental toxicants (bisphenol A, phthalates exposure), systemic pathologies and pathologies that affect the reproductive system as inflammation, infections, and varicocele (99). Although low ROS levels are necessary for several sperm physiological functions, high ROS concentration causes lipid peroxidation, DNA fragmentation, inactivation of enzymes and oxidation of proteins in spermatozoa (100). In particular, OS leads to alterations of sperm motility, affecting flagellar axonemal structure (101) and morphology. Spermatozoa themselves can produce high ROS levels by means of dysregulation of electron transport in the mitochondria, elevated NADPH oxidase activity, or the excessive stimulation of amino acid oxidase action (102). The relationship between sperm morphology and OS can be addressed from two different points of view. First, one of the most frequent defects found in immature spermatozoa is the presence of retained cytoplasm that is not physiologically eliminated during spermiogenesis. In the residual cytoplasm, the NADPH system is activated *via* the hexose-monophosphate shunt and represents a source of electrons for ROS production (103, 104). Second, spermatozoa that reside in an oxidant environment show alterations in motility, morphology, and viability consequent to damage at several levels, as plasma and acrosomal membrane, chromatin and mitochondria (102). A correlation between the presence of ROS and abnormal morphology evaluated by LM (105) was reported and, recently, teratozoospermia was associated with sperm apoptosis, OS and decreased antioxidant capacity of the semen (106). TEM studies underlined a prevalence of immaturity in case of varicocele and necrosis in case of infections/inflammation and in idiopathic infertile patients (20, 33, 107, 108); these pathologies share an inflammatory/OS background.

## Sperm morphology as a monitor of sperm quality in *in vitro* experiments

Human and animal spermatozoa can be used also as an *in vitro* model to test drugs and chemicals and the morphology could be considered, together with motility and vitality, a worthwhile monitor of sperm quality.

A topic debated in the literature refers to the use of antioxidants in male infertility. The antioxidants are essentially administered as dietary supplements to improve human sperm quality (109, 110) even though the real utility of nutraceutical products in male reproductive health is debated (111). *In vivo* studies on animal models can help to understand the effects of a standardized diet on germ cell morphology during spermatogenesis (112).

An interesting research field concerns the *in vitro* treatment of spermatozoa with antioxidants during gamete handling as centrifugations, cryopreservation, procedures in which OS is exacerbated (Supplementary Figure 1). Recently, a general agreement on the protective activity of these compounds, in particular at membrane and acrosomal levels, has been found (113–119). The role of sperm morphology, as a monitor for spermiotoxicity due to different compounds, is also important. For example, Castellini et al. (120) demonstrated that different metals caused different morphological alterations of head sperm membrane in rabbits: arsenic, cadmium, mercury, and platinum were responsible for acrosome damage and formation of microvesicles, arsenic, cadmium, and chromium of large round hole, finally vanadium caused numerous folds in the acrosomal membrane. The role of hexavalent chromium in inducing acrosomal reaction has been confirmed by Yoisungnern et al. (121).

These *in vitro* experiments using the spermatozoon as indicator after a treatment are useful to test compounds showing potential male contraceptive activity. Das et al. (122) demonstrated, by TEM and other techniques, that a compound derived from the plant *Sesbania sesban Merrill*, affected sperm membranes compromising motility and vitality and proposed it as a possible candidate for spermicidal activity. Membrane alterations were observed when the essential oil of *Trachyspermum ammi* (123) and the *Escherichia coli* recombinant sperm immobilizing factor (124) were used in treating human sperm *in vitro*.

## Conclusions

We deeply think that the field of sperm morphology is far from being exhausted and needs other research since this parameter can be still considered a valuable indicator of sperm dysfunction both in basic and clinical research. From a clinical point of view, in case of systematic sperm defects the altered morphology plays a key role in the fertilization failure. More difficult is defining the role of sperm shape in case of non-systematic sperm defects that are responsible for a sperm subpopulation where a particular cell type could have increased chance of fertilization (125). For this purpose, a recent study (126) shows a strong correlation between sperm morphology and expression and methylation status of ten genes, which represent a sort of sperm signature and a new tool for sperm analysis during ARTs and in exploring male infertility. This fascinating hypothesis of “sperm signature” based on molecular and morphological traits could represent the base of further studies to clarify the role of sperm morphology in the clinical outcome.

## Data availability statement

The raw data supporting the conclusions of this article will be made available by the authors, without undue reservation.

## Author contributions

Conceptualization and planning of the original draft: EM and GC. Image editing: RC and DN. Collect data, writing, editing, and revision: EM, GC, CS, DN, and RC. All authors contributed to the article and approved the submitted version.

## Conflict of interest

The authors declare that the research was conducted in the absence of any commercial or financial relationships that could be construed as a potential conflict of interest.

## Publisher's note

All claims expressed in this article are solely those of the authors and do not necessarily represent those of their affiliated organizations, or those of the publisher, the editors and the reviewers. Any product that may be evaluated in this article, or claim that may be made by its manufacturer, is not guaranteed or endorsed by the publisher.

## Supplementary material

Supplementary Figure 1 Scheme representing the role of the morphology studied at transmission electron microscopy level during the *in vitro* treatment of spermatozoa with antioxidants. The oxidative stress induced *in vitro* or by gamete handling such as centrifugations, cryopreservation can cause damage to sperm as acrosome reaction, the use of antioxidant compounds can protect sperm.
