# Supplementary figures and images for: The relevance of sperm morphology in male infertility
Source: Front Reprod Health. 2022 Aug 3;4:945351. doi: 10.3389/frph.2022.945351 (PMC9580829; doi:10.3389/frph.2022.945351)

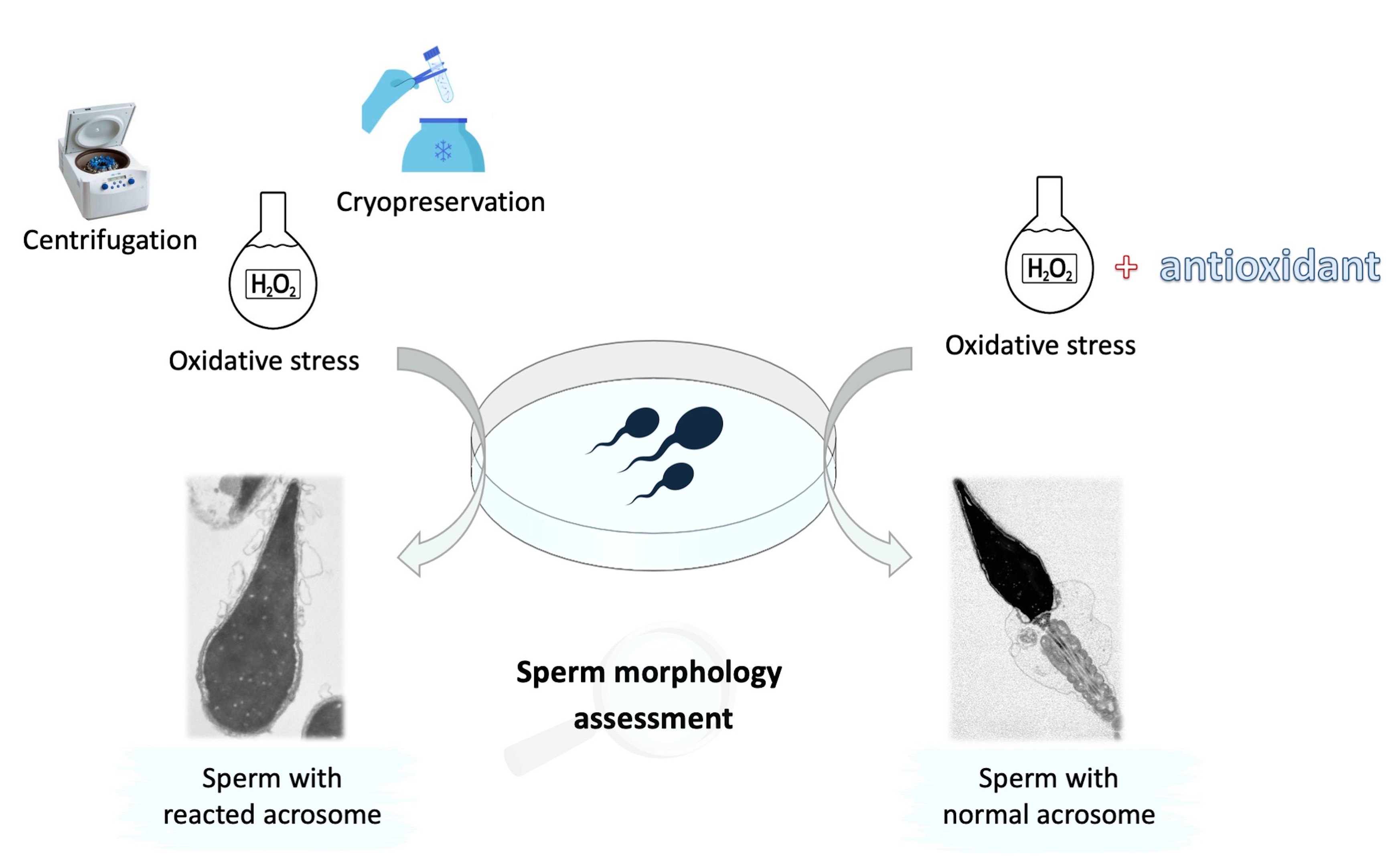

Supplement: Supplementary Figure 1 — Scheme representing the role of the morphology studied at transmission electron microscopy level during the in vitro treatment of spermatozoa with antioxidants. The oxidative stress induced in vitro or by gamete handling such as centrifugations, cryopreservation can cause damage to sperm as acrosome reaction, the use of antioxidant compounds can protect sperm. [file Image_1.JPEG]
